# A randomized, controlled clinical study of low-molecular-weight heparin improving pregnancy outcomes in PCOS women undergoing IVF: study protocol

**DOI:** 10.1186/s13063-023-07877-x

**Published:** 2024-01-02

**Authors:** Ou Huang, Haixia Ding, Dandan Wu, Qing Zhang, Wen Li

**Affiliations:** 1 Department of General Surgery, Comprehensive Breast Health Center, Ruijin Hospital, Shanghai Jiao Tong University School of Medicine, Shanghai, 200025 China; 2 The International Peace Maternity and Child Health Hospital, School of Medicine, Shanghai Jiao Tong University, Shanghai, 200030 China; 3 Shanghai Key Laboratory of Embryo Original Diseases, Shanghai, China

**Keywords:** PCOS, Frozen embryo transfer, Low-molecular-weight heparin, Randomized controlled trial, Ongoing pregnancy rate

## Abstract

**Background:**

Polycystic ovary syndrome (PCOS), an incidence of 10–15% in women of reproductive age, shows sex hormone disorders, luteal insufficiency, and the tendency of placental villus space thrombus. The incidence of early pregnancy loss in women with PCOS is three to eight times higher than that in non-PCOS women. PCOS women were reported in a pre-thrombotic state, which was manifested by accelerated thrombin production, increased PAI-1 activity, and fibrinogen. Other research also found an over-activated state of women with PCOS in immune system. Therefore, changing the prethrombotic state of PCOS through anticoagulation may be a new way to improve the adverse pregnancy outcome of PCOS. Low-molecular-weight heparin (LMWH) is the most common used anticoagulant drug in pregnancy, and it also was proposed for the prevention of recurrent abortion, although the application of LMWH in PCOS population during early pregnancy has not been reported. The objective of this study is to investigate the effect of LMWH on pregnancy outcomes after invitro fertilization-frozen embryo transfer (IVF-FET) in patients with polycystic ovary syndrome.

**Methods:**

A total of 356 PCOS women aged between 20 and 38 years which prepared for IVF followed with FET will be enrolled in the study. The patients, from four different hospitals stratified by age and body mass index (BMI), will be randomly divided into the study group who will be treated with LMWH started on the day of progesterone transformation (hormone therapy) during FET cycle and the control group without additional medicine. Serum or urine hCG test will be given 14 days after embryo transfer to confirm biochemical pregnancy. If pregnancy is positive, LMWH+ hormone therapy/hormone therapy will be continued for another 2 weeks. Transvaginal ultrasonography will be performed 14 days later to confirm intrauterine pregnancy. The primary outcome is the ongoing pregnancy, which is defined as intrauterine live fetus with ultrasound after 12 weeks of gestation.

**Discussion:**

This is the first study protocol to investigate the efficacy of LMWH as an adjuvant drug for IVF-FET outcomes in PCOS women, by comparing differences in ongoing pregnancy rate, clinical pregnancy rate, live birth rate, and early pregnancy loss rate between LMWH group and the control group.

**Trial registration:**

ClinicalTrials.gov ChiCTR2000036527. Registered on August 24, 2020

**Supplementary Information:**

The online version contains supplementary material available at 10.1186/s13063-023-07877-x.

## Background

Polycystic ovary syndrome (PCOS) is one of the most common endocrine disorders in women of reproductive age, characterized by androgen hyperplasia, anovulation, and polycystic ovary, with an incidence of 9–18% during reproductive-aged women [1, 2]. In addition to the mechanism of ovulation disorder, PCOS patients also have the problem of poor endometrial receptivity. Regardless of natural or assisted conception, early pregnancy loss is pervasively among PCOS patients, 3–8 times higher than that of non-PCOS [3]. Adverse pregnancy outcomes not only cause great psychological and physiological harm to patients but also increase the social and economic burden. Therefore, what kind of intervention can improve pregnancy outcomes of PCOS patients has become the urgent task to be solved.

With more attention paid to the high incidence of cardiovascular diseases in PCOS patients, the disorder of coagulation system in PCOS patients has been proved in several studies. A pre-thrombotic state was proposed during PCOS women, which was manifested by increased activity of plasminogen activating inhibitor-1 (PAI-1) and fibrinogen. As a major inhibitor of fibrinolysis, the increase of PAI-1 is associated with low fibrinolysis, suggesting a high risk of thrombosis, while the increase of fibrinogen increases fibrin formation, plasma viscosity, and platelet aggregation, leading to hypercoagulability [4]. Low sex hormone-binding globulin (SHBG) and high insulin may partly explain the body mass index (BMI) independent difference of PAI-1 activity between women with PCOS and control group. It was also reported that the thrombin production rate of PCOS women was faster than that of non-PCOS ones, suggesting a higher risk of thrombosis [5]. Other researchers suggested that PCOS was an autoimmune disease [6], which was associated with a variety of autoimmune diseases, such as systemic lupus erythematosus and Hashimoto’s thyroiditis. Women with PCOS were proved to be involved in increased antibody levels, including antinuclear antibodies, antithyroid antibodies, anti-smooth muscle, anti-histone and anti-double-stranded DNA antibodies, also overactivation of the immune system, and pathophysiology of varying degrees of vascular endothelial damage. It is well known microthrombus formation in uterine embryo and placental circulation is closely related to pregnancy loss including RSA, fetal growth restriction, gestational hypertension, placental abruption, and stillbirth [7]. Therefore, changing the prethrombotic state of PCOS through anticoagulation may provide a new idea for improving pregnancy outcomes of PCOS.

Low-molecular-weight heparin (LMWH) is a glucosaminoglycan with 12~18 sugar units and an average relative molecular weight of 4000~5000, which is produced by enzymatic or chemical depolymerization of ordinary heparin. By binding with antithrombin III (AT-III), LMWH inhibits the activity of thrombin factor Xa and quickly suppresses thrombin formation, achieving anticoagulant effect. Its advantages include of little effect on platelets, rarely causing bleeding tendency, not passing through placenta, no secretion in milk, etc., belonging to FDA: Class B drugs (no reports of fetal malformation have been found). Therefore, it is the preferred anticoagulant drug in pregnancy at present. According to expert consensus published in 2018, LMWH is an effective drug for the treatment of recurrent spontaneous abortion (RSA) caused by antiphospholipid syndrome (APS), prethrombotic state (PTS), and autoimmune diseases [8, 9]. In recent years, some scholars have proposed the application of low-molecular-weight heparin to prevent recurrent abortion. Studies have supported the use of LMWH to increase live birth rate over aspirin in patients with unexplained recurrent pregnancy loss at risk for thrombosis [10, 11]. However, subsequent case-randomized controlled studies suggested that LMWH therapy did not improve unexplained recurrent abortion [12, 13]. Those conflicting results may owe to the heterogeneity of the coagulation status of the subjects and the diversity of the causes of abortion.

In addition, studies have shown other functions of LMWH, including immune regulation, promoting proliferation, invasion, and differentiation of trophoblast cells, inhibiting apoptosis of trophoblast cells, protecting vascular endothelium, and promoting placenta formation.

In conclusion, it is speculated that for PCOS infertility women with prethrombotic state and associated immune dysfunction, LMWH may improve membrane microcirculation during peri-implantation and enhance embryos development*.* However, so far, there are limited data for the administration of LMWH for in vitro fertilization frozen embryo transfer (FET) cycle in PCOS patients. Therefore, a randomized controlled trial is performed to prospectively evaluate the effect of LMWH on pregnancy outcomes of FET cycles, including ongoing pregnancy rate, clinical pregnancy rate, live birth rate, and early pregnancy loss rate.

## Methods

### Research design

This is an open-label randomized controlled superiority trial. Potentially eligible women will be given information about the study before invitro fertilization (IVF) treatment in four different tertiary hospitals: the International Peace Maternity and Child Health Hospital, Second Affiliated Hospital of Naval Medical University, the First Affiliated People’s Hospital of Wenzhou Medical University, and Taizhou Hospital of Zhejiang province. Training is uniformly conducted before the start of the trial to ensure the consistency of procedures in all the centers. The progress is periodically checked by professionals to ensure the uniformity and consistency. The sponsor of this trial is the International Peace Maternity and Child Health Hospital, School of Medicine, Shanghai Jiao Tong University. The research project was approved by the Ethics Committee of The International Peace Maternity and Child Health Hospital and was conducted according to the Declaration of Helsinki. The study was registered on ClinicalTrials.gov (no: ChiCTR2000036527).

**Fig. 1:**
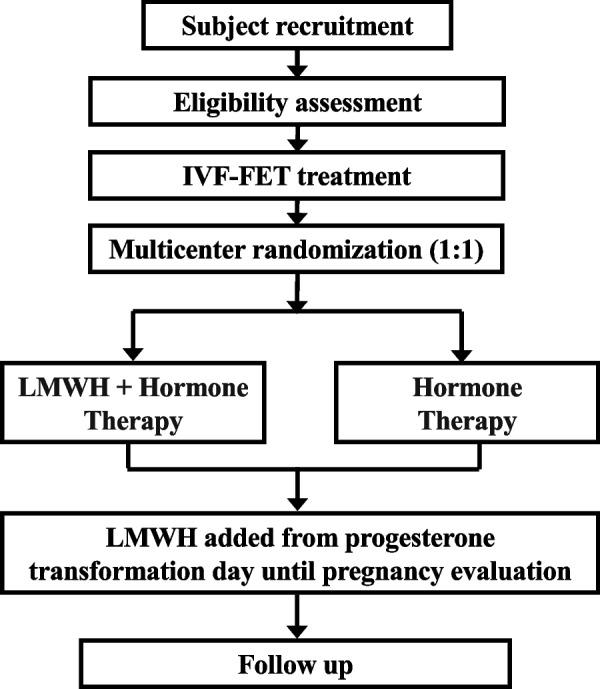
Flowchart of the study

The flow chart of this study is displayed in Fig. 1. A schedule of enrollment, interventions, and assessment is provided as Table 1.

**Table 1:** Schedule of enrollment, interventions, and assessments

Unique form	Study period							
	Enrollment	Intervention phase		Follow-up				
		Ovarian stimulation	Frozen embryo transfer	2 weeks after FET	4 weeks after FET	10 weeks after FET	28 weeks of gestation	Perinatal period
Eligibility screen	𝙓							
Informed consent	𝙓							
Demographic characteristics	𝙓							
Medical/treatment history	𝙓							
Physical exam	𝙓							
Ultrasound	𝙓		𝙓	𝙓	𝙓			
Basic sex hormone	𝙓		𝙓	𝙓	𝙓			
Coagulation function assessment	𝙓		𝙓	𝙓	𝙓			
Safety test	𝙓							
IVF treatment		𝙓						
Randomization			𝙓					
Frozen embryo transfer			𝙓					
Pregnancy evaluation				𝙓	𝙓	𝙓	𝙓	
Perinatal data collection								𝙓

### Recruitment

Recruitment advertisements for the trial were posted on the official platforms of each participating institution. PCOS women undergoing IVF at any one of the four participating institutions will be screened for eligibility. The potential subjects who meet the enrollment criteria will be referred to investigators by their physician on the first day attending IVF department. This trial will be explained by a designated investigator in detail prior to signing informed consent. Written informed consent will be obtained before joining the trial. Participants can decline to join the trial or quit at any time without any consequences for their clinical treatments.

### Inclusion criteria

Patients who met the following inclusion criteria are included in the study:

1. Chinese women aged between 20 and 38 years
2. Meet Rotterdam PCOS standard diagnosis
3. Two of the following three criteria are met: (1) sparse ovulation or anovulation, (2) hyperandrogenemia or clinical manifestations of hyperandrogenism, and (3) ultrasonography performed on 3 to 5 days after bleeding during the menstrual cycle or after progesterone withdraw show both ovaries had ≥ 12 small follicles with a diameter of 2 to 9 mm and/or ovarian volume > 10 mL.
4. No IVF history
5. Willing to cooperate with the research plan and sign the informed consent

### Exclusion criteria used in this study:

1. Other causes of hyperandrogenism, such as thyroid disease congenital adrenal cortical hyperplasia
2. A history of three or more miscarriages or combined with immunogenic infertility
3. Complicated with severe male infertility and immune infertility
4. The presence of uterine lesions: Uterine malformation, adenomyosis, submucosal myoma, or uterine adhesions
5. Prior unilateral oophorectomy
6. Chromosomal abnormalities and need to undergo preimplantation genetic testing (PGT)
7. Have contraindications to assisted reproductive technology
8. Contraindications to drug use: Unexplained liver disease or abnormal liver function (abnormal serum liver enzyme indicators); kidney disease or abnormal renal function; severe anemia; history of deep vein thrombosis and pulmonary embolism history and history of cerebrovascular accident; diagnosed heart disease; a history of cervical, endometrial, or breast cancer; and vaginal bleeding of unknown cause

### Randomization and allocation concealment

PCOS women meeting the inclusion criteria will be recruited and randomized according to an online Central Randomization System stratified by the study sites and women’s age (35 years), supported by Shanghai Clinical Research Center, on the day of progesterone transformation. Participants are assigned in a 1:1 ratio randomly to the study group (LMWH + hormone therapy) or the control group (hormone therapy). LMWH is given daily subcutaneous starting from the progesterone transformation day. The dynamic block method is used to develop the randomization scheme, and the multicenter electronic random system is used to stratify the subjects within the block. Both investigators and participants are unblinded for the allocation of the intervention, while the biostatistician will be blinded to the group allocation.

### Sample size calculation

According to the previous data, the continuous pregnancy rate of female PCOS patients treated with LMWH within 1 week was 45%, while the continuous pregnancy rate of PCOS patients with IVF after routine pre-treatment was about 30%. According to the optimal efficacy experiment, *α* = 0.05, *β* = 0.20, and 10% loss of follow-up and shedding were set. According to the sample size formula, about 178 subjects in each group and 356 subjects should be recruited.

### Adherence

Participants are fully informed that the trial does not involve the increase blood test, and patient visits, as well as the safety and application of the intervention. The attending in charge system improves the relationship between clinicians and participants. Participants can consult the clinician in the clinic at each visit about the treatment regimen and adverse effects if any. Any trial-related questions or discomfort can be communicated with our clinical experts by WeChat platform, and participants can get advice on treatment.

### Interventions

Participants will be subjected to an antagonist regimen of controlled ovulation stimulation, egg extraction, and whole embryo cryopreservation. During the FET cycle, the endometrium will be prepared by hormone therapy. As endometrium thickness is above 8 mm, progesterone will be added preparing for embryos transfer. Clexane 4000 IU (0.4 mL) is given injected daily from the progesterone transformation until hCG determination showing negative or transvaginal ultrasound verifying the presence of intrauterine pregnancy.

Concomitant treatments of immunosuppressants and other drugs that alter blood coagulation condition, such as hydroxychloroquine and warfarin sodium, are not permitted during the trial.

### Outcome measures

The primary outcome will be the ongoing pregnancy rate resulting from the FET cycles, which is defined by the presence of a gestational sac with a fetal heartbeat after 12 weeks of gestation. Secondary outcomes include live birth rate, preterm delivery, pregnancy complications, and early pregnancy loss rate. Live birth rate is defined as the percentage of birth cycles with at least one surviving birth to transplant cycles in the number of transplant cycles. Preterm delivery is defined as a delivery of gestational age of less than 37 weeks. Pregnancy complications mainly refer as preeclampsia (PE) and gestational diabetes (GDM). PE is defined by the combination of gestational hypertension and proteinuria occurring after 20 weeks of gestation. GDM is made when any of the following plasma glucose values are met or exceeded: (1) fasting: 92 mg/dL (5.1 mmol/L); (2) 1 h: 180 mg/dL (10.0 mmol/L); and (3) 2 h: 153 mg/dL (8.5 mmol/L). Early pregnancy loss is defined as loss of the embryo before 12 weeks of gestation, and early pregnancy loss rate refers to the percentage of early pregnancy loss cycles in the number of clinic pregnancy cycles.

### Safety assessment

All subjects will be followed up by medical personnel including a chief physician throughout this study. Although LMWH is the preferred anticoagulant drug for pregnant women, we do not rule out that its use could cause some side effects. The commonly reported adverse reactions of LMWH use were blooding, and less common adverse effects include heparin-induced thrombocytopaenia, osteoporosis, and spontaneous fractures. Patients will be well informed of the potential risks in advance. Follow-up will be performed in the outpatient department at weeks 2, 4, 6, 8, and 10 after FET to record any local and/or systemic reactions during medication and any adverse events defined as below.

#### Adverse events

Adverse events(AEs) are defined as any discomfort or worsening of existing discomfort during the study period (including ovulation induction cycle, FET cycle, and follow-up), regardless of whether associated with the study intervention or not. Discomfort may be symptomatic (e.g., abdominal pain, abdominal distension, chest distress), physical signs (e.g., mobility dullness positive, adnexal mass) or abnormal examination (e.g., laboratory examination, electrocardiograph). Serious adverse events: adverse events occurring during the study period that meet one or more of the following criteria:

1. Severe IVF complications, including severe ovarian hyperstimulation syndrome (OHSS), complete ovarian torsion, and intraperitoneal bleeding. Severe OHSS is diagnosed when ovarian enlargement of ≥ 12 cm is observed and when there is clinical evidence of ascites and/or hydrothorax or breathing difficulties with or without hemoconcentration, severe hypoproteinemia, abnormal liver function, coagulation abnormalities, or diminished renal function.
2. Serious complications of LMWH: Thrombocytopenia, damage of the liver, and kidney function
3. Pregnancy-related complications: Massive bleeding during pregnancy, shock during pregnancy, etc. Adverse events will be reported in the appropriate section of the case report form. It is important that these records include the duration, extent, relationship to the study intervention and/or course of intervention, and any combination therapy.

If serious adverse events occurred, discontinuing or modifying allocated interventions for the participant would be carried out after the participant be fully informed.

### Data collection and monitoring

Data Safety Monitoring Board (DSMB) is consisted of a group of independent experts external to this trial assessing the progress, safety data, to ensure problems will be addressed in an unbiased way by the trial leadership. Besides, in this trial, Clinical Trial Steering Committees undertake the primary responsibility for designing the study, maintaining the quality of study conduct, and ongoing monitoring of individual toxicities and adverse events. Protocol committee will be responsible for protocol setting and correction and also writing.

Baseline characteristics of patients will be collected, including age, BMI, parity, and education levels. Also, detailed IVF process and outcomes will be recorded in time*.* Once a patient is enrolled, a staff team will be prespecified for the follow-up and data collection. Meanwhile, the auditor will conduct data verification and quality check from time to time. The investigator and the monitor work together to ensure information of case report form (CRF) is accurate and clear. Data corrections or additions to CRF must be made by qualified researchers, signed with initial and dated. Data editing, input, and validation are performed continuously and rapidly in the study. Any missing, impossible, or inconsistent data in the CRF will be asked of the investigator via the Data Query Form and then archived for each subject until data cleansing is complete. Clinical research integration platform is used for data entry and management.

The informed consent is compliance with the relevant data protection and privacy laws. All study-related information will be stored securely at the study site. All participant information will be stored in locked file cabinets in areas with limited access. All laboratory specimens and data collection will be identified by a coded identification number to maintain participant confidentiality. All records that contain names or other personal identifiers will be stored separately from study records identified by code number. Participants’ study information will not be released outside of the study without the written permission of the participant. Participants will authorize the collection, use, and publication of the study data by the investigator and those who need the information for the study. Written informed consent will be obtained from patients before study enrollment.

During the trial, protocol amendment will be performed if the most relevant and critical modifications that affect the trial design and data collection procedure occurred. A modified version will be submitted to the Ethics Committee of The International Peace Maternity and Child Health Hospital for ethics approval and to the data monitoring committee. By video or live sessions to ensure that interested parties are known and well understood important protocol changes. The revised protocol would be printed and sent to interested parties.

### Statistical analysis

The study data are collected and managed by nonclinical staff who are responsible for data management in each clinical center. In case of missing values of baseline characteristics, we will analyze them by excluding the missing values, then assign the missing values multiple times, and conduct subsequent analyses to estimate the robustness of the results. For follow-up loss and protocol violation, we will attempt sensitivity analysis to explore the influence of these factors on the trial results.

Baseline characteristics will be described by descriptive analysis, and the balance among groups or subgroups will be assessed by analysis for different kinds of data. Continuous variables with normal distribution are presented as means with standard deviations (SDs) and continuous variables non-normally distributed as medians and interquartile ranges (IQRs). Categorical variables are presented in the form of *n* (%). The result will be analyzed according to the intention-to-treat (ITT) principle. The primary outcome (the ongoing pregnancy rate) and categorical secondary outcomes will be compared between groups by means of log-binomial regression. Adjusted risk ratios (aRRs) and unadjusted risk ratios (RRs), as well as their associated 95% confidence intervals (CIs), will be presented. And the 95% CI of the absolute rate differences (ARDs) will be used to evaluate if the LMWH group is superior to the control group. Continuous data with normal distribution will be analyzed using a two-sample *t*-test, and the Mann-Whitney *U*-test will be used for non-normally distributed data. Subgroup analysis is planned in cases categorized by year (≥ 35 years, < 35 years) and BMI (< 25, ≥ 25). To test the potential interaction between subgroups and treatment effect, we will fit a model with fixed effects to compare the treatment arms with respect to the primary outcome of the ongoing pregnancy rate and binary secondary outcomes (e.g., rates of live birth, preterm delivery, pregnancy complications, and early pregnancy loss). Statistics will be run using SPSS version 21 software (SPSS, Inc., Chicago, IL, USA). *P*-values of less than 0.05 were considered to be statistically significant.

## Discussion

To our knowledge, this is the first study protocol to investigate the efficacy of LMWH as an adjuvant drug for IVF-FET outcomes in PCOS women. PCOS is one of the most important causes of female infertility. Increased risk of embryo implantation failure and early embryo loss rates were revealed by previous studies in patients with PCOS. Hypercoagulability and prethrombotic state might contribute to in PCOS patients’ pregnancy loss. LMWH preventative treatment was empirically used in RSA patients and partly showed positive results.

Our retrospective case-control study showed that significant higher clinic pregnancy rate and livebirth rate in LMWH group (supplemented with LMWH from the day of progesterone transformation) compared with those of control group. LMWH treatment seems to reduce the early pregnancy loss in IVF-FET. Therefore, it is speculated that for PCOS infertility women with prethrombotic state and associated immune dysfunction, LMWH may help to improve membrane microcirculation during peri-implantation and enhance embryos development. However, so far, there are limited data for the administration of LMWH for FET cycle in PCOS patients. Therefore, a randomized controlled trial is designed to prospectively evaluate the effect of LMWH on the IVF outcomes of FET cycles, including ongoing pregnancy rate, clinical pregnancy rate, live birth rate, and early pregnancy loss rate.

The strengths of the present study include that this is a multicenter, central dynamic randomized trial. Age, BMI, and subcenters were designed as block factors for minimizing bias. There are also some limitations: For LMWH is given subcutaneous, although good adherence in patients, it is not suitable for blind design. The nature of the LMWH treatment is open to treating physicians but blind to embryologists, laboratory technicians, and follow-ups of this study; the study population is Han of ethnic, and further studies are necessary to verify our conclusions correction in other ethnic populations, with varied genotype and phenotype.

### Trial status and peer review

The first participant was recruited on 12th March 2021, and the approximate date of recruitment will be completed on 11th March 2024. The follow-up is ongoing, and the expected data collection will be completed in December 2024. This trial protocol is version 3.0 (1 March 2021). This study is externally peer-reviewed.

## Supplementary Information

**Additional file 1.** SPIRIT Checklist for Trials.

## Data Availability

All data generated during and/or analyzed during the study will be presented within the manuscript, and other detailed data are available from the corresponding author on reasonable request.

## Abbreviations

APS: Antiphospholipid syndrome
AT-III: Antithrombin III
BMI: Body mass index
CRF: Case report form
hCG: Human chorionic gonadotropin
IQRs: Interquartile ranges
ITT: Intention to treat
IVF-ET: In vitro fertilization embryo transfer
FET: Frozen embryo transfer
LMWH: Low-molecular-weight heparin
OHSS: Ovarian hyperstimulation syndrome
PAI-1: Plasminogen activating inhibitor-1
PCOS: Polycystic ovary syndrome
PGT: Preimplantation genetic testing
PP: Per protocol
PTS: Prethrombotic state
RCT: Randomized controlled trial
RSA: Recurrent spontaneous abortion
SDs: Standard deviations

## References

[1] March WA, Moore VM, Willson KJ, Phillips DI, Norman RJ, Davies MJ. The prevalence of polycystic ovary syndrome in a community sample assessed under contrasting diagnostic criteria. Hum Reprod. 2010;25(2):544–51. 10.1093/humrep/dep399. 19910321 10.1093/humrep/dep399

[2] Yildiz BO, Bozdag G, Yapici Z, Esinler I, Yarali H. Prevalence, phenotype and cardiometabolic risk of polycystic ovary syndrome under different diagnostic criteria. Hum Reprod. 2012;27(10):3067–73. 10.1093/humrep/des232. 22777527 10.1093/humrep/des232

[3] Essah PA, Cheang KI, Nestler JE. The pathophysiology of miscarriage in women with polycystic ovary syndrome. Review and proposed hypothesis of mechanisms involved. Hormones (Athens). 2004;3(4):221–7. 10.14310/horm.2002.11130. 16982596 10.14310/horm.2002.11130

[4] Manneras-Holm L, Baghaei F, Holm G, et al. Coagulation and fibrinolytic disturbances in women with polycystic ovary syndrome. J Clin Endocrinol Metab. 2011;96(4):1068–76. 10.1210/jc.2010-2279. 21252248 10.1210/jc.2010-2279

[5] de Mendonca-Louzeiro MR, Annichino-Bizzacchi JM, Magna LA, Quaino SK, Benetti-Pinto CL. Faster thrombin generation in women with polycystic ovary syndrome compared with healthy controls matched for age and body mass index. Fertil Steril. 2013;99(6):1786–90. 10.1016/j.fertnstert.2013.01.105. 23415973 10.1016/j.fertnstert.2013.01.105

[6] Hepsen S, Karakose M, Cakal E, et al. The assessment of thyroid autoantibody levels in euthyroid patients with polycystic ovary syndrome. J Turk Ger Gynecol Assoc. 2018;19(4):215–9. 10.4274/jtgga.2018.0001. 29699958 10.4274/jtgga.2018.0001 PMC6250086

[7] Stewart CJR. Endometrial intravascular thrombi are typically associated with shedding but may be the sentinel feature of an underlying thrombotic disorder. Histopathol. 2020;76(6):919–22. 10.1111/his.14074. 10.1111/his.14074 31984504

[8] Pleguezuelo DE, Cabrera-Marante O, Abad M, et al. Anti-phosphatidylserine/prothrombin antibodies in healthy women with unexplained recurrent pregnancy loss. J Clin Med. 2021;10(10) 10.3390/jcm10102094. 10.3390/jcm10102094 PMC8152729 34068095

[9] Hamulyak EN, Scheres LJJ, Goddijn M, Middeldorp S. Antithrombotic therapy to prevent recurrent pregnancy loss in antiphospholipid syndrome-what is the evidence? J Thromb Haemost. 2021;19(5):1174–85. 10.1111/jth.15290. 33687789 10.1111/jth.15290 PMC8252114

[10] Gris JC, Mercier E, Quere I, et al. Low-molecular-weight heparin versus low-dose aspirin in women with one fetal loss and a constitutional thrombophilic disorder. Blood. 2004;103(10):3695–9. 10.1182/blood-2003-12-4250. 14739212 10.1182/blood-2003-12-4250

[11] Hamulyak EN, Scheres LJ, Marijnen MC, Goddijn M, Middeldorp S. Aspirin or heparin or both for improving pregnancy outcomes in women with persistent antiphospholipid antibodies and recurrent pregnancy loss. Cochrane Database Syst Rev. 2020;5(5):CD012852. 10.1002/14651858.CD012852.pub2. 32358837 10.1002/14651858.CD012852.pub2 PMC7195627

[12] Kaandorp SP, Goddijn M, van der Post JA, et al. Aspirin plus heparin or aspirin alone in women with recurrent miscarriage. N Engl J Med. 2010;362(17):1586–96. 10.1056/NEJMoa1000641. 20335572 10.1056/NEJMoa1000641

[13] Schleussner E, Petroff D. Low-molecular-weight heparin for women with unexplained recurrent pregnancy loss. Ann Intern Med. 2015;163(6):485. 10.7326/L15-5137-3. 26370020 10.7326/L15-5137-3

